# Progesterone exerts a neuroprotective action in a Parkinson’s disease human cell model through membrane progesterone receptor α (mPRα/PAQR7)

**DOI:** 10.3389/fendo.2023.1125962

**Published:** 2023-03-10

**Authors:** Luca F. Castelnovo, Peter Thomas

**Affiliations:** Marine Science Institute, The University of Texas at Austin, Port Aransas, TX, United States

**Keywords:** Parkinson’s disease, membrane progesterone receptor α, neuroprotection, PI3K-AKT, ERK

## Abstract

Parkinson’s disease (PD) is the second most common neurodegenerative disease worldwide, and current treatment options are unsatisfactory on the long term. Several studies suggest a potential neuroprotective action by female hormones, especially estrogens. The potential role of progestogens, however, is less defined, and no studies have investigated the potential involvement of membrane progesterone receptors (mPRs). In the present study, the putative neuroprotective role for mPRs was investigated in SH-SY5Y cells, using two established pharmacological treatments for cellular PD models, 6-hydroxydopamine (6-OHDA) and 1-methyl-4-phenylpyridinium (MPP+). Our results show that both the physiologic agonist progesterone and the specific mPR agonist Org OD 02-0 were effective in reducing SH-SY5Y cell death induced by 6-OHDA and MPP^+^, whereas the nuclear PR agonist promegestone (R5020) and the GABA_A_ receptor agonist muscimol were ineffective. Experiments performed with gene silencing technology and selective pharmacological agonists showed that mPRα is the isoform responsible for the neuroprotective effects we observed. Further experiments showed that the PI3K-AKT and MAP kinase signaling pathways are involved in the mPRα-mediated progestogen neuroprotective action in SH-SY5Y cells. These findings suggest that mPRα could play a neuroprotective role in PD pathology and may be a promising target for the development of therapeutic strategies for PD prevention or management.

## Introduction

Parkinson’s disease (PD) is the second most common neurodegenerative disorder. Due to aging of the world’s population, its prevalence is predicted to reach 9 million people worldwide by 2030 (1, 2). The disease is characterized by the progressive degeneration of dopaminergic neurons in the *substantia nigra* projecting to the striatum, the presence of Lewy bodies, and microgliosis (3, 4). Current treatments aim to control the motor symptoms, with no effects on the progression of the degenerative process, and lose efficacy with time (5). PD incidence and prevalence is higher in men than women (6–8). Moreover, based on epidemiological studies, it has been proposed that PD symptoms may have a later onset in women, possibly because of the neuroprotective effect of female sex hormones earlier in life (9–11). Therefore, it has been hypothesized that female sex hormones may play a neuroprotective role. Both epidemiological studies and animal models support a possible neuroprotective role of estrogens (9–15). Several *in vivo* animal studies, with different PD experimental models have also shown that progesterone (P4) may have neuroprotective action in dopaminergic neurons. In particular, several studies showed that P4 can be neuroprotective in the presence of 1-methyl-4-phenyl-1,2,3,6-tetrahydropyridine (MPTP), in a common *in vivo* model of PD damage, if administered before MPTP treatment (16–19). There is some evidence suggesting that P4 may also be potentially used as a treatment after the onset of PD symptoms. Indeed, P4 counteracts MPTP toxicity in male mice if administered within a short time window after the insult (20). Moreover, it bolsters dopaminergic differentiation in mouse embryonic stem cells undergoing an *in vitro* differentiation protocol, which may be beneficial in PD treatment (21). However, the putative neuroprotective activity of P4 in human cell models of PD and the progestogen receptors mediating this P4 action have not been investigated.

Membrane progesterone receptors (mPRs) are members of the progestin and adipoQ receptor (PAQR) family (22, 23), presenting five isoforms: mPRα/PAQR7, mPRβ/PAQR8, mPRγ/PAQR5 (24, 25), mPRδ/PAQR6 and mPRϵ/PAQR9 (26). These receptors were initially characterized in reproductive tissues, but are known to exert several activities also in non-reproductive tissues, including the nervous system (27). Moreover, mPRs have been reported to mediate progestogen neuroprotective actions in different neuronal cell line models (28–30). In particular, the specific mPR agonist Org OD 02-0 (02–0) (31) displayed neuroprotective activity in SH-SY5Y cells in a model of cell starvation (30). SH-SY5Y cells are a neuroblastoma cell line which show catecholaminergic characteristics. This cell line is widely used in PD research, with different protocols used for cell culture and different strategies employed to induce PD-like conditions (32). In this study we investigated the putative neuroprotective role of mPRα using two chemicals commonly used to mimic PD damage in cell cultures, 6-hydroxydopamine (6-OHDA) and MPTP’s active metabolite, 1-methyl-4-phenylpyridinium (MPP^+^) (32). mPRα neuroprotective activity to decrease cell death was examined following pharmacological activation of PD, and the specificity of mPRα’s action was confirmed with specific agonists and gene silencing studies. The intracellular mechanisms underlying the putative neuroprotective activity of mPRα were also investigated.

## Materials and methods

### Cell culture and pharmacological treatments

The SH-SY5Y neuronal cell line was obtained from American-type Cell Culture Collection (ATCC, Manassas, VA, USA). The medium used for cell culture was composed of 85% minimum essential medium, α modification (α-MEM, Lonza, Morristown, NJ, USA), with 2 nM L-glutamine (Gibco, Waltham, MA, USA) supplementation, and 15% fetal bovine serum (FBS, Corning Inc., Corning, NY, USA).

For the majority of experiments cells underwent overnight incubation in α-MEM supplemented with 2 nM L-glutamine (serum-free condition) before treatment for 24h with various pharmacological agents. For Western blot experiments requiring 6-OHDA pretreatment, 6-OHDA was added to the serum-free medium for the overnight incubation prior to progestogen treatment. Phosphorylation of ERK and AKT was assessed by Western blot analysis. Several time points were considered in preliminary experiments for 02-0 treatments (5,10 and 30 min, data not shown). The 10 min treatment was selected since it showed the stronger response. 6-OHDA (Tocris, Minneapolis, MN, USA) and MPP^+^ iodide (Sigma-Aldrich, St. Louis, MO, USA) were used to mimic PD damage in SH-SY5Y cells. The specific mPR agonist 02-0 (Axon Medchem, Groningen, Netherlands), the natural agonist P4 (Steraloids, Newport, RI, USA), the specific PR agonist R5020 (Steraloids) and the specific GABA-A receptor agonist muscimol (Tocris) were used to assess the contribution of different progestogen receptors. The specific ERK inhibitor AZD 6244 (Stemcell Technologies, Vancouver, BC, Canada), the specific PI3K inhibitor Wortmannin (EMD Millipore, Burlington, MA, USA) and the specific AKT inhibitor ML-9 (EMD Millipore) were used to investigate the activation of intracellular signaling pathways. The final concentrations to be used for treatments were either determined in a series of preliminary experiments or obtained from the literature. The different drugs were used at the following concentrations (when the concentration was obtained from the literature, a specific reference is provided in the following list): 6-OHDA 50 μM (33); MPP^+^ 750 nM; 02-0 100 nM (30, 34); P4 100 nM (30); R5020 100 nM (30, 34); muscimol 100 μM (35); AZD 6244 1 μM (36); Wortmannin 50 nM (34); ML-9 15 μM (34). A comparison between the concentrations used for these chemicals and their EC_50_/IC_50_ is presented in **Table S1**. Unless they were soluble in serum-free medium (Mus, 6-OHDA and MPP^+^), pharmacological treatment results were compared with those of their vehicle (Veh) solvents: EtOH (02-0, P4, R5020), DMSO (AZD 6244, Wortmannin, ML-9), or a combination of the two.

### Hoechst assay

The Hoechst assay was performed as previously described (30). SH-SY-5Y cells were grown on coverslips inside multiwell plates and then underwent 20 min fixation with 4% formaldehyde (Thermo-Fisher Scientific, Waltham, MA, USA). After a rinse with phosphate buffer saline (PBS, Thermo-Fisher Scientific), cells were incubated with Hoechst 33342 (Tocris) 1 μg/mL for 5 min. The cells were then rinsed and observed with an Eclipse Ti2 microscope (Nikon, Tokyo, Japan). Cell death was assessed by changes in chromatin morphology, nuclei with compacted chromatin were counted as Hoechst positive. At least 20 images from different coverslips were acquired for each condition, with each acquired image representing a single sample. The determination of the percentage of Hoechst positive nuclei was performed with the ImageJ software (NIH, Bethesda, MD, USA).

### RNA extraction and quantitative real time PCR (RT-qPCR)

The extraction of total RNA from samples was performed with TriReagent (Invitrogen, Carlsbad, CA, USA), following the manufacturer’s instructions, and quantification was performed with a Nanodrop 2000c UV spectrophotometer (Thermo-Fisher Scientific, Waltham, MA, USA). Samples were treated with the TURBO DNA-free™ kit (Thermo-Fisher Scientific) for the removal of any DNA contamination. Every sample in the RT-qPCR experiments was run in triplicate, with 10 ng of total RNA in each reaction. The RT-qPCR reaction was performed with a Mastercycler^®^ RealPlex 2 (Eppendorf North America, Hauppauge, NY, USA), using the Luna Universal One-Step RT-qPCR kit (New England Biolabs, Ipswich, MA, USA). The primers used in these experiments have been previously published (34). Testing was performed to make sure every primer had an efficiency included between 90 and 105%. No-template controls were used as negative controls for all the experiments. The primers used are listed in **Table S2**.

### Protein extraction and Western blot analysis

Cells were scraped in PBS, and then suspended in RIPA buffer (Sigma-Aldrich), with added protease and phosphatase inhibitors (EMD Millipore). They were then homogenized and centrifuged for 5 minutes at 800 g. The Western blot experiments were performed using the supernatant from this centrifugation. Protein samples were denatured at 100°C for 3 min. 20 μg of total protein samples were loaded into each well of SDS-PAGE 4-20% precast gels (Bio-Rad, Hercules, CA, USA) and underwent electrophoretic separation at 200 V for 50 minutes in running buffer. Proteins were then transferred to Hybond nitrocellulose membranes (Bio-Rad) by electro-blotting. The blocking phase was performed with 5% not-fat dry milk (Bio-Rad) in 0.1% PBS-Tween 20 (Sigma-Aldrich), followed by incubation with the primary antibodies diluted in the same solution. The following antibodies, all from Cell Signaling Technologies, were used: mouse anti-Akt 1:500; rabbit anti-pAkt Ser473 1:200; rabbit anti-pERK 1/2 1:500; mouse anti-ERK 1/2 1:200. After a rinse in PBS, membranes were incubated in PBS-Tween 20 0.1% containing suitable IRDye secondary antibodies. The antibodies, both from LI-COR Biosciences (Lincoln, NE, USA), were used at 1:7000 dilution: goat anti-rabbit IRDye 800; goat anti-mouse IRDye 680. After washing, the IR signal was detected with an Odyssey scanner (LI-COR Biosciences).

### Silence RNA assay

Human Silencer Select PAQR7 siRNA, Silencer Select Negative Control #2 siRNA and Lipofectamine RNAiMAX were purchased from Invitrogen, and the silencing procedure was performed following the manufacturer’s instructions. Briefly, the day before the start of the silencing procedure, cells were plated at 50% confluence. The following day, the specific PAQR7 siRNA and the negative control (NC) oligo were dissolved in OptiMEM (Invitrogen). They were then mixed with Lipofectamine, previously dissolved in OptiMEM, and incubated for 20 minutes. The PAQR7 siRNA and the NC oligo were then transferred to the samples at a final concentration of 10 nM and incubated for 48 hours. The medium was then removed, and the cells were treated as described above. Silencing efficiency was tested by RT-qPCR.

### Data analysis and statistics

All the described experiments were repeated at least three times. Statistical analyses was performed using Prism 5.0 (GraphPad, San Diego, CA, USA). Different statistical analyses were employed, depending on the experimental design: two-tailed unpaired Student’s t-test, one-way ANOVA, or two-way ANOVA. When a one-way ANOVA was used, single conditions were then compared among them with Tukey’s multiple comparison test. When two-way ANOVA was used, single conditions were compared by post-hoc multiple comparison of means, using the Bonferroni correction. P values < 0.05 were considered significant. All results are expressed as mean ± standard error of the mean (SEM).

## Results

### 02-0 and P4 treatments are neuroprotective in SH-SY5Y cells

**Figure 1:**
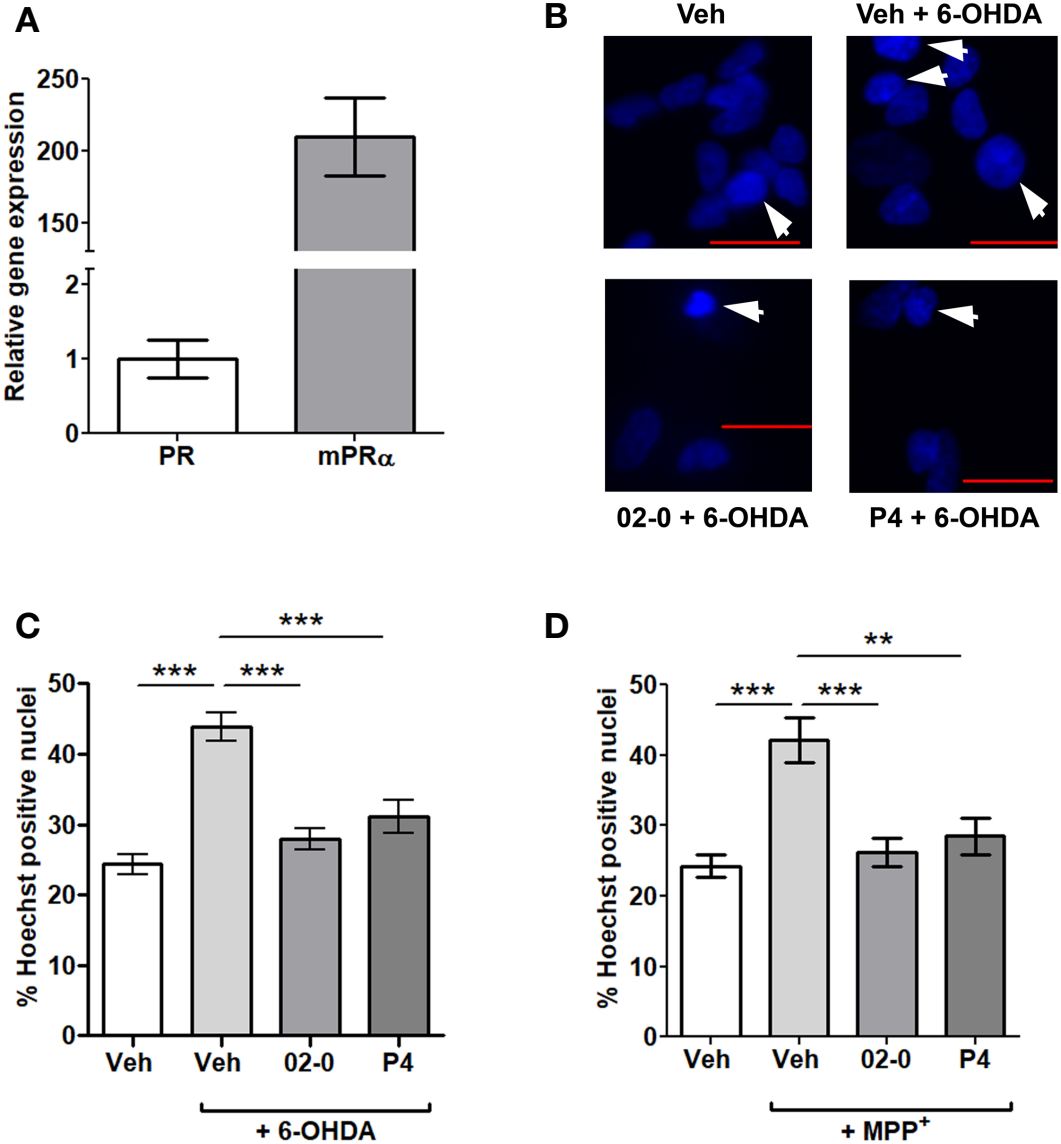
PR and mPRα expression and the effects of P4 and 02-0 treatments on 6-OHDA- and MPP^+^-mediated cell death in SH-SY5Y cells. **(A)** Gene expression of PR and mPRα in SH-SY5Y cells. N=6. **(B)** Representative images of SH-SY5Y cell nuclei labeled with Hoechst 33342 in the 6-OHDA experiment. Nuclei in which chromatin appears compacted and were counted as positive are indicated by an arrow. Scale bar: 20 μm. **(C)** Assessment of cell death in SH-SY5Y cells in the Hoechst assay following 24h Veh, 100 nM 02-0 and 100 nM P4 treatments in the presence or absence of 50µM 6-OHDA. N=18. One-way ANOVA results: p<0.0001. Tukey’s multiple comparisons test results: ***: p<0.001. **(D)** Assessment of cell death in SH-SY5Y cells following Veh, 100 nM 02-0 and 100 nM P4 24 h treatments in presence or absence of 750 nM MPP^+^. N=18. One-way ANOVA results: p<0.0001. Tukey’s multiple comparisons test results: **: p<0.01: ***: p<0.001.

The gene expression of the nuclear P4 receptor (PR) and mPRα were determined by RT-qPCR. The average Ct value for PR was 33.82 ± 0.39, while the average Ct for mPRα was 25.90 ± 0.22. The calculated mPRα expression was 200 times higher than that of the PR in SH-SY5Y cells (**Figure 1A**).

The putative neuroprotective actions of the specific mPR agonist 02-0 and the physiological mPR ligand P4 to decrease cell death in SH-SY5Y cells were tested. 6-OHDA and MPP^+^ 24 h treatments significantly increased cell mortality, as determined by the Hoechst assay (**Figures 1B–D**). Both 100 nM 02-0 and P4 were effective in protecting SH-SY5Y cells from toxicity, significantly reducing the fraction of Hoechst- positive nuclei in cells treated with the neurotoxic chemicals to levels similar to those in Veh-treated cells not exposed to the chemicals (**Figures 1C, D**). Therefore, mPRs likely have a neuroprotective action in SH-SY5Y cells.

### Progestogen neuroprotective activity is mediated through mPRα

**Figure 2:**
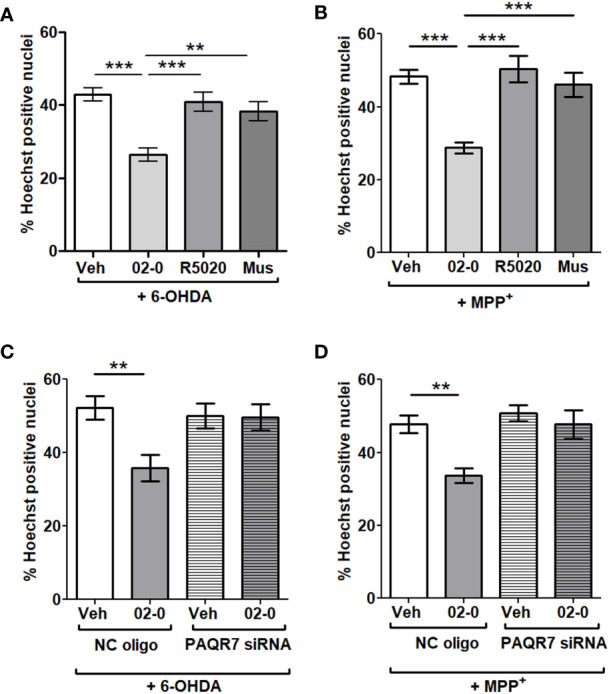
Investigation of the possible contribution of different progestogen receptors in P4 neuroprotection in SH-SY5Y cells **(A)** Assessment of cell death in SH-SY5Y cells following Veh, 100 nM 02-0, 100 nM R5020 and 100 μM Mus (muscimol) 24 h treatments in presence 50 μM 6-OHDA. N=18. One-way ANOVA results: p<0.0001. Tukey’s multiple comparisons test results: **: p<0.01: ***: p<0.001. **(B)** Assessment of cell death in SH-SY5Y cells following Veh, 100 nM 02-0, 100 nM R5020 and 100 μM Mus (muscimol) 24 h treatments in presence of 750 nM MPP^+^. N=18. ***: p<0.001. **(C)** Assessment of cell death in SH-SY5Y cells following Veh and 100 nM 02-0 24 h treatments in presence of 50 μM 6-OHDA and following PAQR7 (mPRα) gene silencing. N=18. Two-way ANOVA results: Interaction: p=0.0274, Treatment: p=0.0196, Silencing: p=0.1006. Bonferroni’s post-test results: **: p<0.01. **(D)** Assessment of cell death in SH-SY5Y cells following Veh and 100 nM 02-0 24 h treatments in presence of 750 nM MPP^+^ and following PAQR7 gene silencing. N=18. Two-way ANOVA results: Interaction: p=0.0514, Treatment: p=0.0033, Silencing: p=0.0036. Bonferroni’s post-test results: **: p<0.01.

We then investigated the possible contribution of different 02-0 and P4-binding receptors to the neuroprotective activity described above. Treatment with 100nM 02-0 significantly decreased cell death as observed previously. However, both the PR specific agonist R5020 (100nM) and the GABA-A receptor specific agonist muscimol (100µM) were ineffective and did not show a neuroprotective action during 6-OHDA or MPP^+^ 24 h incubations in the Hoechst assay, suggesting these two receptors are not involved in the neuroprotective action of P4 (**Figures 2A, B**). Once we determined that neuroprotection was mPR-mediated, we performed mPRα siRNA experiments to determine if the neuroprotection is mediated by this mPR isoform. The RT-qPCR assessment of silencing efficiency revealed that mPRα expression was reduced by 79.9% (**Figure S1**). The specific PAQR7 siRNA completely abolished the 02-0 protective effect on cell viability in presence of both neurotoxic chemicals (**Figures 2C, D**), which suggests that mPRα is the isoform promoting neuroprotection in SH-SY5Y cells.

### mPRα differently activates the ERK and PI3K-AKT signaling pathways in SH-SY5Y cells

**Figure 3:**
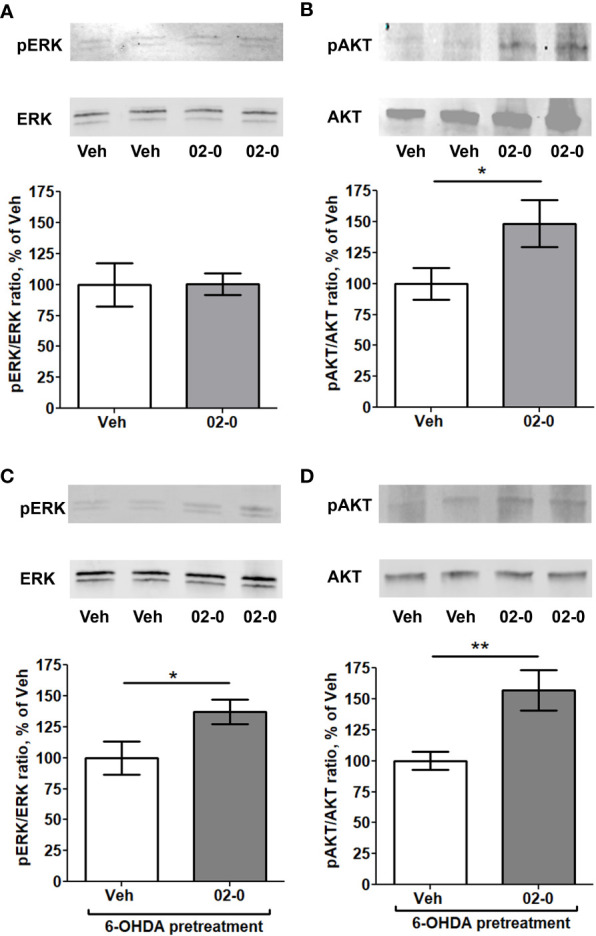
Activation of intracellular signaling pathways by 02-0 in SH-SY5Y cells **(A)** Western blot for ERK 1/2 and pERK 1/2 in SH-SY5Y cells after 10 min treatment with Veh or 100 nM 02-0, with representative blot showing bands at 42 and 44 KDa. N=8. Student’s t-test results: p>0.05. **(B)** Western blot for AKT and pAKT in SH-SY5Y cells after 10 min treatment with Veh or 100 nM 02-0, with representative blot showing bands at 62 KDa. N=8. Student’s t-test results: *: p<0.05. **(C)** Western blot for ERK 1/2 and pERK 1/2 in SH-SY5Y cells after 10 min treatment with Veh or 100 nM 02-0 following overnight pretreatment with 50 μM 6-OHDA, with representative blot showing bands at 42 and 44 KDa. N=8. Student’s t-test results: *: p<0.05. **(D)** Western blot for AKT and pAKT in SH-SY5Y cells after 10 min treatment with Veh or 100 nM 02-0 following overnight pretreatment with 50 μM 6-OHDA, with representative blot showing bands at 62 KDa. N=8. Student’s t-test results: **: p<0.01.

We next examined the signaling pathways involved in the neuroprotective activity of mPRα. We first tested the effects of 100nM 02-0 treatment of SH-SY5Y cells on two intracellular pathways often linked to mPRα activity in other human tissues (34, 37–40): the ERK and PI3K-AKT pathways. To this end, we analyzed by Western blot the effect of 02-0 treatments on the phosphorylation levels of ERK kinases 1/2 and the AKT kinase. Following 10 min treatment, 02-0 did not affect ERK phosphorylation (**Figure 3A**), but it significantly increased AKT phosphorylation (**Figure 3B**). We then repeated these experiments after pretreating SH-SY5Y cells overnight with 6-OHDA to model a PD condition. In this experiment, phosphorylation of both ERK and AKT was significantly increased after 02-0 treatment (**Figures 3C, D**). Therefore, both these signaling pathways can be activated by mPRα in these cells and may be involved in its neuroprotective action.

### Both ERK and PI3K-AKT signaling pathways mediate mPRα neuroprotection

**Figure 4:**
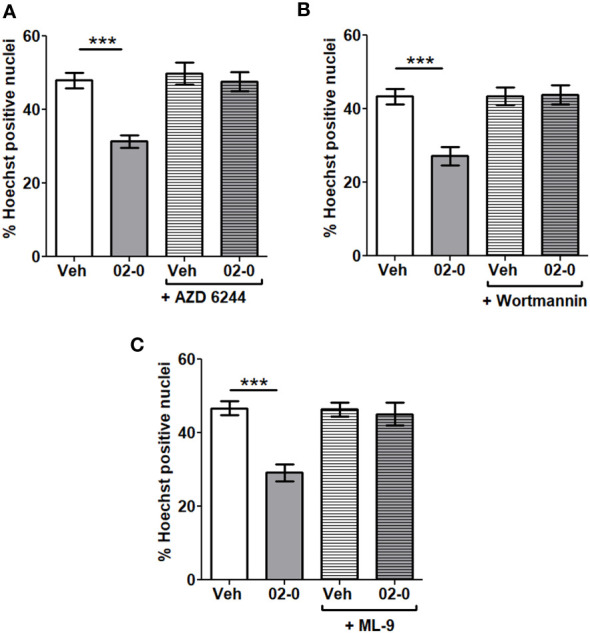
Involvement of signaling pathways in mPRα-mediated neuroprotection **(A)** Assessment of cell death in SH-SY5Y cells following Veh and 100 nM 02-0 24 h treatments in the presence of 50 μM 6-OHDA, with or without 1μM AZD 6244. N=18. Two-way ANOVA results: Interaction: p=0.0039, Treatment: p=0.0002, Inhibitor: p=0.0004. Bonferroni’s post-test results: ***: p<0.001. **(B)** Assessment of cell death in SH-SY5Y cells following Veh and 100 nM 02-0 24 h treatments in presence of 50 μM 6-OHDA, with or without 50 nM Wortmannin. N=18 Two-way ANOVA results: Interaction: p=0.0009, Treatment: p=0.0015, Inhibitor: p=0.0008. Bonferroni’s post-test results: ***: p<0.001. **(C)** Assessment of cell death in SH-SY5Y cells following Veh and 100 nM 02-0 24 h treatments in presence of 50 μM 6-OHDA, with or without 15 μM ML-9. N=18. Two-way ANOVA results: Interaction: p=0.0009, Treatment: p=0.0001, Inhibitor: p=0.0014. Bonferroni’s post-test results: ***: p<0.001.

We performed studies with specific inhibitors of the above-mentioned signaling pathways to verify their roles in mPRα-mediated neuroprotective activity. The specific ERK inhibitor AZD 6244 (**Figure 4A**), the PI3K inhibitor Wortmannin (**Figure 4B**) and the AKT inhibitor ML-9 (**Figure 4C**) were used in a series of experiments when cells were treated for 24 h in the presence of 6-OHDA. All inhibitors proved effective in completely blocking the 02-0 protective effect on SH-SY5Y cell viability in the Hoechst assay. These results suggest both the ERK and the PI3K-AKT signaling pathways are involved in promoting mPRα-mediated neuroprotective activity.

## Discussion

The results presented in this study show that P4 is neuroprotective in a cell model of PD through mPRα activation. The intracellular mechanism underlying mPRα activity involves the ERK and PI3K-AKT signaling pathways.

Non-differentiated SH-SY5Y cells were used for this study. Several differentiation protocols, mostly involving incubation with retinoic acid, have been proposed for this model in PD research. However, none of them is widely accepted, and a recent systematic review found that a no differentiation protocol was used in 81.5% of studies on SH-SY5Y cells as a PD model (32).

The comparison of mPRα and PR gene expression show that mPRα expression is 200 times higher than PR, likely making it the main mediator of P4 activity in this cell model. PR and mPRα were previously reported to be expressed in SH-SY5Y cells (30, 41, 42), but their expression levels were never compared. Our findings show that 02-0 and P4 are both effective in reducing SH-SY5Y cell death induced by 6-OHDA and MPP^+^ treatments. Both chemicals cause cell toxicity by causing oxidative stress. MPP^+^ increases the intracellular concentration of reactive oxygen species (ROS) by inhibiting complex I of the electron transport chain, thus impairing mitochondrial function. Moreover, it causes dopamine mobilization towards the cytoplasm, further increasing ROS production (43). 6-OHDA enters the cell through dopaminergic or catecholaminergic transporters. It then accumulates inside the cell, causing increased formation of ROS and catecholamine quinones, leading to cell death (44). P4 was previously shown to reduce ROS formation in ovarian and endometrial cancer cells (45) and to increase the expression and activity of the electron transport chain complex IV, thus reducing ROS formation, in the brain of ovariectomized rats (46). Therefore, even though mPRα influence on oxidative stress has not been characterized yet, it is reasonable to hypothesize that a modulation of ROS production may be involved in mPRα-mediated neuroprotection. This hypothesis requires further investigation.

In SH-SY5Y cells, 02-0 was previously shown to reduce cell death in a cell starvation model (30), and P4 was reported to be neuroprotective in models of pyroptosis (47) and neurological complications of HIV (41). Moreover, the P4 active metabolite allopregnanolone had protective activity in a model of Alzheimer’s disease following β-amyloid treatment (48). P4 was also neuroprotective in *in vivo* PD models, in particular following MPTP treatment (19, 20). Progestogens proved effective in eliciting neuroprotective effects also in other neuronal cell models. For example, P4 and allopregnanolone were neuroprotective, through mPR activation, in rodent GT1-7 and H19-7 neuronal cells in models of cell starvation (26, 28, 29). It should be noted that mPR activation was also shown to mediate neuroregenerative effects in peripheral glial cells (37, 49, 50). Since mPRs are also expressed in central nervous system glial cells (51, 52) and their expression in these cells in the brain is upregulated following traumatic brain injury (51), it can be hypothesized that mPRs in glial cells may be neuroprotective in PD. This hypothesis will need to be investigated in the future. Even taking in consideration the neuroprotective effects of progestogens described above, this is to our knowledge the first study showing P4 as a potential neuroprotective agent in an established human cell model of PD.

The pharmacological treatment and siRNA experiments show that mPRα is the receptor mediating progestogen neuroprotective action in SH-SY5Y cells following 6-OHDA and MPP^+^ treatments. As discussed above, mPRα and PR are expressed in SH-SY5Y cells, with the former being predominant. GABA-A receptor subunits were previously reported to be expressed at very low levels in SH-SY5Y cells, with the exception of the β3 subunit which was highly expressed (48). Muscimol treatments confirmed that the GABA-A receptor is not involved in the neuroprotective activity we observed. mPRβ was reported to be the most highly expressed mPR isoform in SH-SY5Y cells (30). However, the present results with PAQR7 siRNA clearly show that mPRα is the dominant isoform in mediating protective effects, as often observed in other human cell models (34, 39, 40).

Western blot and inhibitor studies clearly show that the ERK and PI3K-AKT signaling pathways are involved in the neuroprotective action mediated by mPRα. ERK activation only occurred following 6-OHDA pretreatment, suggesting it may be a specific response to cell damage. As mentioned above, both signaling pathways are known mediators of mPR activity in different human cell models (34, 37–40). Activation of both signaling pathways has been recently reported to be beneficial in different PD models (53–58). Our results also support the neuroprotective role of the ERK signaling pathway, whereas in several other studies the ERK pathway was also linked to possible worsening of PD damage by increasing oxidative stress and inflammation (59, 60). P4 treatment was not effective in activating these pathways in the striatum in an *in vivo* mouse model (20). This difference may be due to interspecies differences or involvement of other cell types, such as glial cells, in the mice striatum.

Taken together, our findings show that mPRα is neuroprotective in a human cell model of PD through the activation of the ERK and PI3K-AKT signaling pathways. These results will need to be confirmed in an *in vivo* model of PD to increase their translational potential. Nonetheless, these findings open the way for further investigation of mPRα as a potential target for progestogen therapies aimed at PD prevention or management.

## Data availability statement

The raw data supporting the conclusions of this article will be made available by the authors, without undue reservation.

## Author contributions

Both authors contributed to the study conception and design. L performed the experiments, wrote the original draft, and prepared the figures. P obtained funding, supervised the project, and revised the manuscript. Both authors approve the final version of the manuscript.
