# Supplementary figures and images for: Progesterone exerts a neuroprotective action in a Parkinson’s disease human cell model through membrane progesterone receptor α (mPRα/PAQR7)
Source: Front Endocrinol (Lausanne). 2023 Mar 10;14:1125962. doi: 10.3389/fendo.2023.1125962 (PMC10036350; doi:10.3389/fendo.2023.1125962)

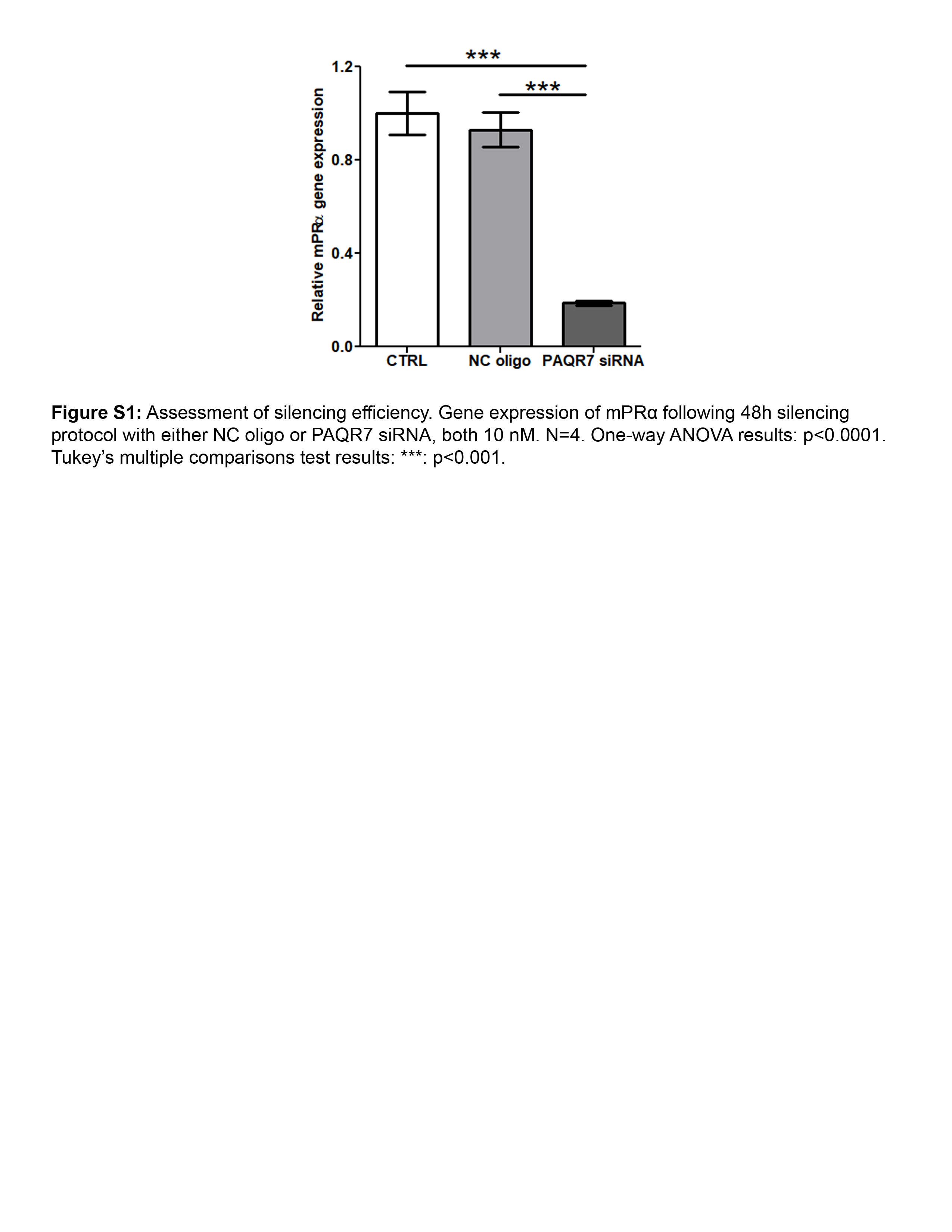

Supplement: Supplementary file 1 [file Image_1.tif]
