# Supplementary material for: Progesterone exerts a neuroprotective action in a Parkinson’s disease human cell model through membrane progesterone receptor α (mPRα/PAQR7)
Source: Front Endocrinol (Lausanne). 2023 Mar 10;14:1125962. doi: 10.3389/fendo.2023.1125962 (PMC10036350; doi:10.3389/fendo.2023.1125962)
Supplement: Supplementary file 2 [file Table_1.pdf]

Table S1 – List of chemicals, with used concentration and published activity/affinity values.

Chemical	Used concentration	Published activity/binding value	Type of value and reference
6-OHDA	50 μM	118 μM	EC_{50}, (1)
MPP^{+}	750 nM	> 4 mM*	EC_{50}, (2)
02-0	100 nM	33.9 nM	IC_{50}, (3)
P4	100 nM	87.4 nM	IC_{50}, (3)
R5020	100 nM	8.90 nM	pIC_{50}, (3)
Mus	100 μM	0.7 μM	IC_{50}, (4)
AZD 6244	1 μM	14 nM	IC_{50}, manufacturer's website
Wortmannin	50 nM	5 nM	IC_{50}, manufacturer's website
ML-9	15 μM	3.8 μM	Ki, manufacturer's website

* Deduced from Figure 1A of the cited article.

References

1. Kang X, Qiu J, Li Q, Bell KA, Du Y, Jung DW, Lee JY, Hao J, Jiang J. Cyclooxygenase-2 contributes to oxidopamine-mediated neuronal inflammation and injury via the prostaglandin E2 receptor EP2 subtype. *Sci Rep* (2017) 7:9459. doi: 10.1038/s41598-017-09528-z
2. Zhao M, Chen J, Mao K, She H, Ren Y, Gui C, Wu X, Zou F, Li W. Mitochondrial calcium dysfunction contributes to autophagic cell death induced by MPP+ via AMPK pathway. *Biochem Biophys Res Commun* (2019) 509:390–394. doi: 10.1016/j.bbrc.2018.12.148
3. Kelder J, Azevedo R, Pang Y, de Vlieg J, Dong J, Thomas P. Comparison between steroid binding to membrane progesterone receptor α (mPRα) and to nuclear progesterone receptor: Correlation with physicochemical properties assessed by comparative molecular field analysis and identification of mPRα-specific agonists. *Steroids* (2010) 75:314–322. doi: 10.1016/j.steroids.2010.01.010
4. Newell JG, Dunn SMJ. Functional Consequences of the Loss of High Affinity Agonist Binding to γ-Aminobutyric Acid Type A Receptors. *J Biol Chem* (2002) 277:21423–21430. doi: 10.1074/jbc.M110312200
