# Supplementary material for: Progesterone exerts a neuroprotective action in a Parkinson’s disease human cell model through membrane progesterone receptor α (mPRα/PAQR7)
Source: Front Endocrinol (Lausanne). 2023 Mar 10;14:1125962. doi: 10.3389/fendo.2023.1125962 (PMC10036350; doi:10.3389/fendo.2023.1125962)
Supplement: Supplementary file 3 [file Table_2.pdf]

Table S2 – List of primers used for RT-qPCR experiments.

Gene (ID)	Forward primer (5'-3')	Reverse primer (5'-3')
mPRα (164091)	CTGAAGTTTGCCTGACACCA	AATAGAAGCGCCAGGTCTGA
PR (5241)	GCATGGTCCTTGGAGGTCGAAAA	TCTGGCTTAGGGCTTGGCTT
GAPDH (2597)	TGACTTCAACAGCGACACCCA	GACAAAGTGGTCGTTGAGGGCA
